# PP66 International Horizon Scanning Initiative (IHSI) : Time to Review the Need for Stand-Alone National Horizon Scanning?

**DOI:** 10.1017/S026646232400237X

**Published:** 2025-01-07

**Authors:** Heather Eames, Marie Harte, Caitríona Ní Choitir, Laura McCullagh, Lesley Tilson, Roisin Adams

## Abstract

**Introduction:**

Ireland is a member of the International Horizon Scanning Initiative (IHSI) and has access to the full horizon for medicines across multiple disease areas and specialized reports on high-impact technologies within disease areas (IHSI High Impact Reports [HIRs]). Health technology developers (HTDs) provide a notification to HTA agencies and payers on intent to seek reimbursement for a technology in the preceding two years.

**Methods:**

Information on technologies submitted to the national HTA agency in 2023 was selected for comparison to information in the IHSI database. The IHSI system detects products prior to regulatory approval. The national notification system (NNS) can receive information post-licensing. We examined how many products were received in the NNS that were either in the regulatory process or licensed in the EU. We sought to compare key information in the NNS and the IHSI database to identify areas of duplication. Areas where information from NNS could complement the IHSI database were highlighted. HIRs were not included in this assessment.

**Results:**

In 2023, 20 percent of products submitted by HTDs via the NNS were already licensed. To compare the information provided in IHSI and the NNS, a sample of 35 technologies common to both systems was selected. The IHSI and the NNS contain multiple data fields (154 in IHSI and 72 in NNS) across domains including information on product characteristics, regulatory information, clinical trial data, and cost. The exceptional fields specific to the Irish context were compassionate access scheme availability in Ireland, Ireland-specific patient population estimates, and local pricing and reimbursement proposals.

**Conclusions:**

Receipt of information from HTDs remains important, particularly country-specific information. There are efficiencies in identifying technologies via an independent horizon scanning system, not reliant on submissions by HTDs. Such a system could facilitate joint assessment, joint early advice, and joint problem-solving between countries. Joint efforts can also facilitate broader scope on how horizon scanning can be used to increase health system preparedness.

